# Understanding the Mechanism for Adsorption of Pb(II) Ions by Cu-BTC Metal–Organic Frameworks

**DOI:** 10.3390/molecules28145443

**Published:** 2023-07-16

**Authors:** Joanna N. Weyrich, John R. Mason, Ekaterina A. Bazilevskaya, Hongwei Yang

**Affiliations:** 1 Department of Chemistry, Widener University, One University Place, Chester, PA 19013, USA; j.nozomi.w@gmail.com; 2 Department of Chemistry and Biochemistry, University of Delaware, 102 Brown Laboratory, Newark, DE 19716, USA; johnrmason1999@gmail.com; 3 Department of Ecosystem Science and Management, The Pennsylvania State University, 409 Agricultural Sciences and Industries Building, University Park, PA 16802, USA; eab204@psu.edu

**Keywords:** Cu-BTC, metal–organic frameworks, adsorption, heavy metal ions, Pb(II)

## Abstract

With the growing population, industrialization, and agriculture, water contamination not only affects people but entire ecosystems. Metal–organic frameworks (MOFs), because of their large surface area and porosity, show great potential as adsorbents for removing pollutants, such as heavy metals, from contaminated water. The current research aims at examining copper (II) benzene-1,3,5-tricarboxylate (Cu-BTC) MOFs and understanding the mechanism for their adsorption of Pb(II) from aqueous solution. The Cu-BTC samples were characterized using FTIR and XRD, and their surface area and porosity were determined based on N_2_ adsorption isotherms. The concentration of Pb(II) in the solutions was measured using atomic absorption spectroscopy (AAS). Both kinetic and equilibrium adsorption data were collected and then analyzed using numerical models. The analyses led to the findings that the limiting steps in the adsorption of Pb(II) on Cu-BTC are (a) pore diffusion of Pb(II) and (b) the availability of the active sites on Cu-BTC MOFs. It was further revealed that the former step is more dominant in the adsorption of Pb(II) when the lead concentration is low. The latter step, which is directly proportional to the surface areas of the MOFs, affects the adsorption to a greater extent when the lead concentration is high. The results also show that adsorption of Pb(II) ions on Cu-BTC is mainly a multi-layer heterogeneous process.

## 1. Introduction

Metal–organic frameworks (MOFs) are porous crystalline materials made up of a network of organic linkers coordinated to metal ions or clusters [1]. The invention of MOFs can be dated to the 1990s, MOF-2: Zn(1,4-benzendicarboxylate)(H_2_O) or Zn(BDC)(H_2_O) being one of the very first MOFs successfully synthesized during that time [2]. Today there are hundreds of different known MOFs. Researchers have found that many of these structures exhibit permanent porosity, which was absent from any previously known porous molecular crystal. Their unique properties of unparalleled porosity and surface area can be attributed to the absence of dead volume. MOFs are also highly versatile due to the fact that their building components can be varied in size, kind, and geometry [3,4]. Other useful properties of MOFs include high thermal stability typically ranging from 250 to 500 °C, high chemical stability in certain structures, and acid/base resistance [5]. All these properties make MOFs considered for many applications.

With the ever-growing problem of water pollution due to human-made waste and unpredictable fluctuations in the environment in the wake of the earth’s rising population, searching for efficient methods for wastewater treatment has become an urgent need alongside with preventing clean water from being polluted in the first place. One group of pollutants in wastewater is emerging organic contaminants (EOCs), specifically organic dyes [6]. Rapidly growing industrialization generates a sizeable amount of dye-contaminated wastewater from industries such as printing, textiles, food, and cosmetics. Dyes present in water pose a threat to human and wildlife health with 17–20% of dyes being responsible for water pollution. Another group of pollutants commonly found in water is heavy metals such as Cr^6+^, Pb^2+^, Cd^2+^, and Hg^2+^ [7]. Heavy metals are a serious problem in that they are indigestive, meaning they accumulate easily in the human body and in the environment. This leads to complications to human beings, such as liver damage or kidney failure, as well as to other living organisms.

MOFs pose a promising solution as selective adsorbents for wastewater treatment [8,9], since they allow for rapid removal, easy separation, and industrial scale synthesis. In recent years, efforts have been made to identify new MOFs and improve existing MOFs for such applications. For example, a variety of copper-based [10,11], iron-based [12,13], chromium-based [14,15], and zinc-based [16,17] MOFs were reported in the literatures to be effective as selective adsorbents in the adsorption of targeted adsorbates from aqueous solutions, including EOCs and/or heavy metal ions. Work has also been done to enhance the adsorption performance of the MOFs by functionalizing the MOFs with desirable groups [18,19,20], such as amino and thiol, as well as by incorporating magnetic cores inside the MOFs [21,22,23]. In many of those studies, however, focus was placed on synthesizing the MOFs, as well as examining and comparing their adsorption performance. There is only a very limited number of studies [11,13,19,20] where an attempt was made to address the adsorption mechanism in detail. Understanding the mechanism, however, is a challenging but important issue, since gaining such insight would facilitate the effort of improving the adsorption performance of the MOFs for wastewater treatment and other desired applications.

The current study focuses on copper (II) benzene-1,3,5-tricarboxylate (denoted by Cu-BTC), one of the most important copper-based MOFs used for many adsorption applications. Cu-BTC MOFs were tested against varying concentrations of lead solutions to determine their capacity for adsorbing lead and the removal rate of lead. In an effort to understand the mechanism for the adsorption of Pb(II) ions on Cu-BTC, the kinetic results of the system were first examined. This led to the findings that the limiting steps in the adsorption of Pb(II) ions on Cu-BTC are determined by (a) pore diffusion of Pb(II) ions and (b) the availability of the active sites on Cu-BTC MOFs. Such findings were further supported by equilibrium data analysis. It was revealed that the adsorption on Cu-BTC MOFs is limited by the diffusion of Pb(II) ions in the pore structure of the MOFs when the lead concentration is low. When the concentration is high, however, the adsorption of Pb(II) ions is affected by the surface areas of the MOFs, which directly relates to the number of active adsorption sites. These results shed light on how to improve adsorptive performance of Cu-BTC and other MOFs in wastewater treatment and other adsorption-based applications.

## 2. Results and Discussion

### 2.1. Characterization of Cu-BTC MOFs

#### 2.1.1. IR Analysis

**Figure 1:**
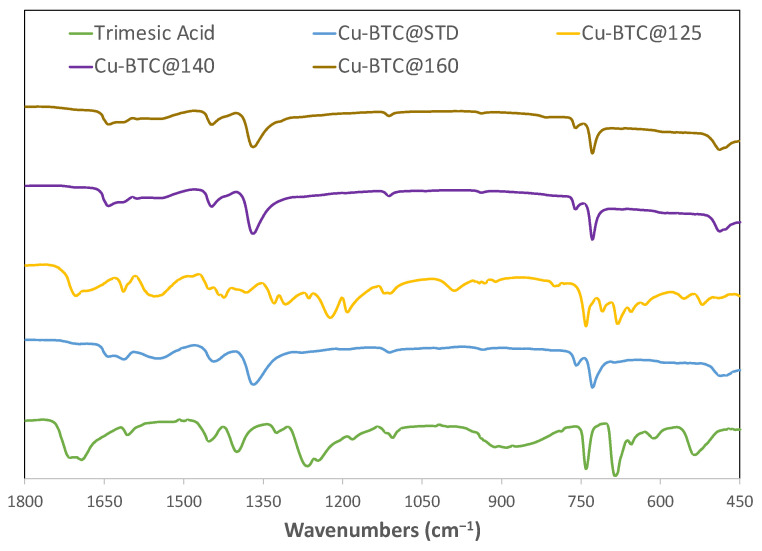
IR spectra of starting reagent trimesic acid, standard Cu-BTC, and Cu-BTC samples synthesized by microwave at temperatures of 125 °C, 140 °C, and 160 °C, respectively.

Cu-BTC samples were characterized by analyzing the IR spectra of the starting reagents and comparing them to the as-synthesized products, as shown in Figure 1. It also includes the Cu-BTC@STD sample, which was Cu-BTC MOFs directly acquired from Sigma Aldrich.

The IR spectrum results support that the samples microwaved at 140 °C and above fully reacted to form Cu-BTC, since these samples showed an identical spectrum to that of the Cu-BTC@STD sample. For instance, both Cu-BTC@140 and Cu-BTC@160 displayed a medium to weak asymmetrical COO^−^ peak around 1650 cm^−1^ along with a strong peak around 1370 cm^−1^ due to the symmetrical COO^−^ stretch. It has been shown [23] that the strong carbonyl frequency near 1730 cm^−1^, commonly found in organic acids such as trimesic acid, vanishes in their salts and is replaced by two bands, one located between 1610 and 1550 cm^−1^ and another in the region 1300–1420 cm^−1^. It is believed that when deprotonation of carbonyl group gives a carboxylate anion COO^−^, resonance is possible between the two C–O bonds, leading to the disappearance of the C=O bands. Two bands at 1650 cm^−1^ and 1370 cm^−1^, as illustrated in Figure 1, were the results of asymmetric and symmetric stretching of a carboxylate anion such as COO^−^. Another band in the spectra of Cu-BTC@140 and Cu-BTC@160 was the doublet around 750 cm^−1^, which could be assigned to the Cu–O stretching vibration. All these characteristic bond structures found in these two samples matched those in the Cu-BTC@STD sample, which confirms the formation of Cu-BTC MOFs via microwave synthesis at temperatures of 140 °C and above.

Included also in Figure 1 is the third sample, Cu-BTC@125, the IR spectrum of which showed a mix of the results from Cu-BTC MOFs and trimesic acid, one of the starting reagents. It indicated an incomplete reaction at such a temperature. The C=O band found with trimesic acid near 1730 cm^−l^ remained visible in the spectrum of this sample. In addition, the C–O signal around 1220 cm^−1^ was believed to stem from trimesic acid. There appeared to still be the Cu-O doublet, but it was weaker and overshadowed by the strong bands at 740 and 680 cm^−1^ that are also found in trimesic acid. All this information supports that the reactions were not fully carried out in this trial, and that there may have been significant traces of one of the starting reagents present.

#### 2.1.2. XRD Analysis

**Figure 2:**
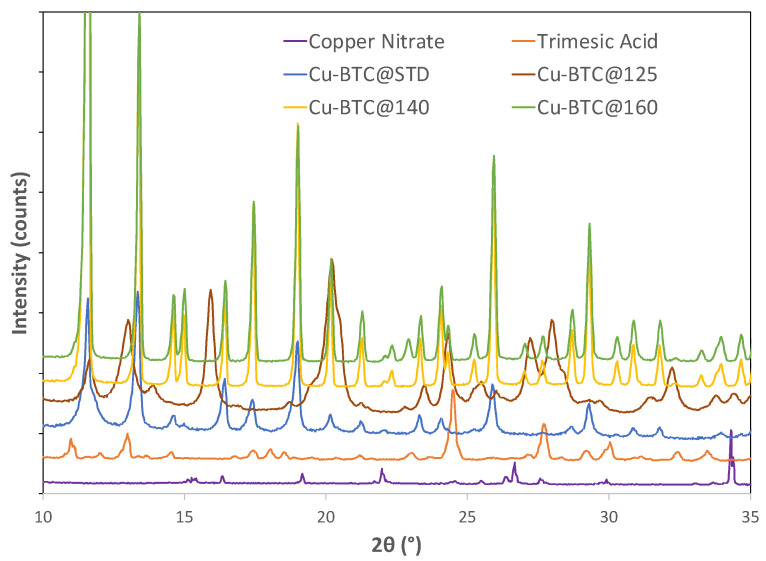
XRD of starting reagents copper nitrate and trimesic acid, standard Cu-BTC, and Cu-BTC samples synthesized by microwave at temperatures of 125 °C, 140 °C, and 160 °C, respectively.

X-ray diffraction (XRD) was performed to further examine the identity of the samples synthesized at various temperatures. The Cu-BTC@STD sample was also included to facilitate the examination. For Cu-BTC@140 and Cu-BTC@160, three pronounced peaks located at 2θ = 11.7°, 13.4°, and 19.0° were observed in their XRD patterns, as illustrated in Figure 2. These characteristic peaks matched what was observed for the Cu-BTC@STD sample as well as what was reported in other studies of Cu-BTC MOFs [10,24,25]. Coupled with the IR results discussed in Section 2.1.1, it could be confirmed that the successful formation of Cu-BTC structures was achieved by microwave syntheses when they were conducted at 140 °C or above.

Compared with Cu-BTC@140 and Cu-BTC@160, on the other hand, the XRD patterns of Cu-BTC@125 contained additional prominent peaks such as those at 2θ = 8.1°, 13.0°, and 15.9°. These peaks could not be attributed to Cu-BTC structures, indicating that microwave synthesis at 125 °C did not lead to the complete formation of Cu-BTC structures. However, there was no clear evidence that such peaks could be attributed to either the ligand or the metal salt. Since the current study was mainly focused on the adsorption property of Cu-BTC MOFs, no further effort was made at this stage to identify the above-mentioned peaks.

#### 2.1.3. N_2_ Adsorption Isotherm Analysis

**Figure 3:**
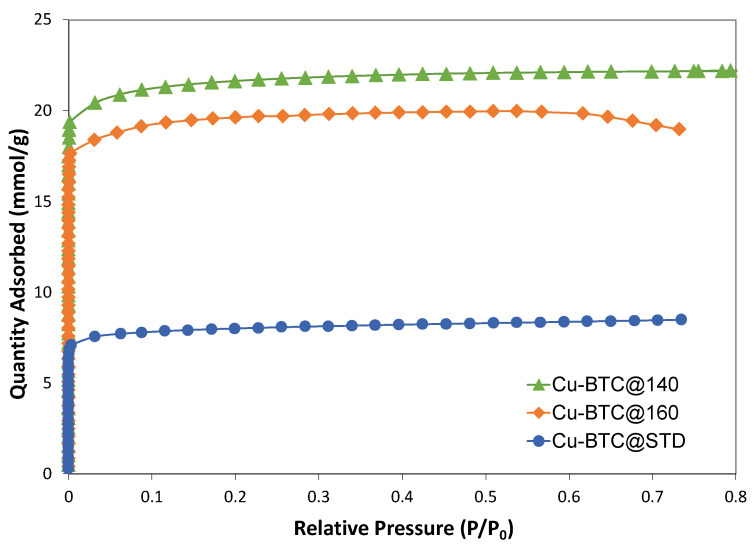
N_2_ adsorption isotherms of standard Cu-BTC, and Cu-BTC samples synthesized by microwave at temperatures of 140 °C and 160 °C, respectively.

It is well known that the porous properties of the adsorbent could play an essential role in the adsorption processes. Therefore, the N_2_ adsorption isotherms were collected at −196 °C to examine the porous properties of the as-synthesized Cu-BTC samples before their performance of adsorbing Pb(II) ions was measured. Figure 3 contains such isotherms for the Cu-BTC samples, which were prepared at 140 °C and 160 °C. It also includes the Cu-BTC@STD sample. All adsorption isotherms showed a steep uptake in adsorption at a very low pressure of *P*/*P*_0_, and then a plateau followed. According to the IUPAC classification [26], these adsorption curves showed the characteristics of a Type-I isotherm, clearly indicative of the dominant presence of micropores in the Cu-BTC samples. Micropores are those pores with widths not exceeding about 2 nm. Such a narrow space enhanced the adsorbent–adsorbate (Cu-BTC and N_2_) interactions, thus enabling the filling of N_2_ in Cu-BTC even at a very low pressure.

Based on the multipoint BET equation, the specific surface areas (SSA) of the Cu-BTC samples were calculated, and the results are included in Table 1. The as-synthesized Cu-BTC samples, including Cu-BTC@140 and Cu-BTC@160, showed specific surface areas ranging from 1700 to 1900 m^2^/g. On the other hand, the Cu-BTC@STD sample had a surface area of about 700 m^2^/g, which was much lower than that of the Cu-BTC samples synthesized by microwave. Using the t-Plot method, further assessment was made to differentiate between micropore areas and external surface areas, since it is believed that these two types of areas in the Cu-BTC samples are likely to be different in affecting the adsorption of Pb(II) ions from aqueous solution. The results in Table 1 show that all the Cu-BTC samples, including the standard one, had a percentage of micropore areas close to 90%. Such a high percentage of internal surface areas is common among porous materials such as MOFs, which have a large number of internal but open pores. As a result, a large portion of the surface areas in such materials stem from the internal surfaces of those pores. In addition to the specific surface areas, the micropore volume and the pore size were also calculated for this series of samples based on the N_2_ adsorption isotherms, as shown in Table 1 as well. The micropore volume among these samples was in line with what was seen for their surface areas. In terms of the pore size, the DFT calculation results shown in Table 1 were largely consistent with what was expected for Cu-BTC MOFs, which have a bimodal pore size distribution [25,27]. The larger pores were approximately 9 Å in diameter, while the smaller ones were close to 5 Å.

### 2.2. Adsorption Mechanism of Pb(II) Ions on Cu-BTC MOFs

An adsorption process is generally believed to involve two major steps [28]. First is the transport of adsorbates to the exterior of adsorbents. For non-porous adsorbents, such mass transport is typically achieved by so-called “film diffusion”, where adsorbates travel across the liquid film surrounding the adsorbent external surface. For porous absorbents, adsorbate diffusion also occurs within the pores of the adsorbents. This is often referred to as “pore diffusion” or “intraparticle diffusion”. The second step in an adsorption process involves the binding of adsorbates to the adsorption sites on the surface of adsorbents. To study adsorption mechanism, in essence, is to examine these two steps for the given adsorption. It is a common practice that this is being achieved by conducting kinetic and equilibrium studies for the adsorption of interest.

Since Cu-BTC samples in the current study had a high percentage of internal surface, as discussed above, pore diffusion was believed to the main factor in affecting the transport of Pb(II) ions inside the structures. The binding of adsorbates to the active adsorption sites is a complex process and is influenced by a variety of factors. The current study focused on two important factors: (a) the availability of active adsorption sites on the surface of Cu-BTC MOFs, and (b) the nature of these active adsorption sites in terms of their homogeneity/heterogeneity.

#### 2.2.1. Adsorption Kinetic Study

**Figure 4:**
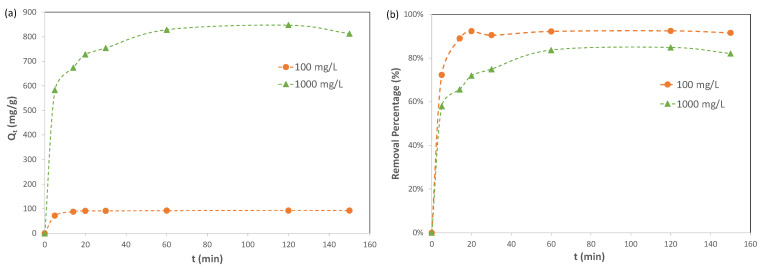
(**a**) Adsorption capacity and (**b**) removal % of Pb(II) by Cu-BTC@STD at various times (*C*_0_ = 100 mg/L and 1000 mg/L).

Adsorption kinetics describes the rate of adsorbate uptake on an adsorbent. In the current study, the adsorption kinetics of Pb(II) ions on Cu-BTC MOFs were investigated for two lead solutions with initial concentrations of Pb(II) ions of 100 mg/L and 1000 mg/L, respectively. The results are included in Figure 4. For the 100 mg/L solution, the adsorption reached equilibrium at 20 min, and the adsorption capacity of Pb(II) ions on Cu-BTC MOFs at equilibrium, *q_e_*, had an averaged value of 91.98 mg/g, equivalent to 92% removal. For the 1000 mg/L solution, the results indicated that the adsorption took a longer time to reach equilibrium, namely, 60 min. However, Cu-BTC MOFs displayed a much higher adsorption capacity of Pb(II) ions at 829.6 mg/g in this solution, while the removal percent was lower, with a value of 83%.

Exploring the adsorption mechanism using kinetic data is common practice in the literature [10,11,12,14,15,17,19], and certain mathematic models are employed to fit the kinetic data. Equations (1) and (2) represents two of the commonly used models, namely, the “pseudo first order” model and the “pseudo second order” model.

(1) $$\ln\left(q_{e}-q_{t}\right)=\ln q_{e}-k_{1}t$$

(2) $$\frac{t}{q_{t}}=\frac{1}{k_{2}q_{e}{}^{2}}+\frac{t}{q_{e}}$$

where $q_{e}$ (mg/g) is the adsorption capacity at equilibrium, and $q_{t}$ (mg/g) is the adsorption capacity at a given adsorption time *t* (min). $k_{1}$ (min^−1^) and $k_{2}$ (g/mg/min) are the rate constants of the pseudo first order adsorption and the pseudo second order adsorption, respectively.

**Figure 5:**
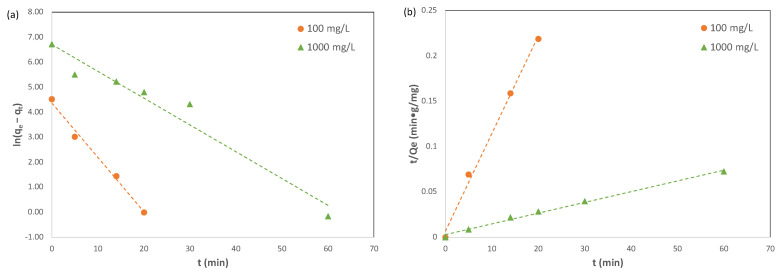
The kinetic data fitting with (**a**) pseudo first order and (**b**) pseudo second order for the adsorption of Pb(II) on Cu-BTC@STD (*C*_0_ = 100 mg/L and 1000 mg/L).

Figure 5 shows the plots of the kinetic data for the adsorption of Pb(II) on Cu-BTC using the pseudo first order and the pseudo second order models, and the corresponding results are summarized in Table 2. It is noteworthy that only pre-equilibrium data points were used for the plots, which included those from 0 to 20 min for the 100 mg/L solution and those from 0 to 60 min for the 1000 mg/L solution. Such steps were taken because a close inspection of the kinetic data fitting practice [29,30] indicated that the inclusion of post-equilibrium data would introduce a methodological bias. As a result, the pseudo second order kinetics model has been widely and unfairly favored as the dominant model.

For both solutions being studied, the results shown in Figure 5 and in Table 2 indicated that the pseudo second order model was still favored over the pseudo first order model to describe the adsorption kinetics of Pb(II) ions on Cu-BTC MOFs. This was based on the comparison made for the coefficient of determination, or R^2^, between these two models. For the 1000 mg/L solution, the R^2^ value was clearly favored for the pseudo second order model, though the R^2^ value for the 100 mg/L solution was only slightly favored for the pseudo second order model. In addition to the R^2^ values, the pseudo second order model also predicted the adsorption capacities (*q_e_*) of 93.46 mg/g and 833.3 mg/g for the two solutions, respectively, which were closer to the experimental values (91.98 mg/g and 829.6 mg/g) than those derived from the pseudo first order model (78.58 mg/g and 814.4 mg/g). Therefore, the pseudo second order model is more suitable to describe the adsorption kinetics of Pb(II) ions on Cu-BTC MOFs.

As described above, the second step in an adsorption process can be characterized in the context of binding between adsorbates and adsorbents. Based on the nature of such interactions, adsorption is generally classified as either physisorption (characteristic of weak van der Waals forces) or chemisorption (characteristic of covalent bonding). In many published works [11,12,14,17], pseudo first order and pseudo second order kinetic models were used as main tools to identify these two different types of adsorption. Claims were made based on the assumption that if the pseudo first order model displays a better fit for the experimental results, then adsorption is a physical process, while if the pseudo second order model fits better, then it is a chemical adsorption. In the current study, caution was taken for interpreting the kinetic results in this manner. This is because (a) the two models as expressed by Equations (1) and (2) were obtained from empirical differential equations, and there was lack of clear evidence that they were derived based on theories of adsorbate–adsorbent interaction; and (b) even though some known chemisorption cases in the literature [8,13] are described well by the pseudo second order model, the opposite is not always true. With such caution being taken, we used these two mathematic models to examine the adsorption based on the fundamentals of kinetic theories, that is, the limiting steps in the adsorption could be identified from the kinetic data.

The adsorption and desorption of a solute A in a solution can be represented by an interface reaction as follows:

(3) $$A_{\left(l\right)}+S_{\left(s\right)}\;\begin{array}{c} k_{a}\\ \Leftrightarrow \\ k_{d} \end{array}\;AS_{\left(s\right)}$$

where $k_{a}$ and $k_{d}$ are the adsorption and desorption rate constants, respectively, the subscripts (*l*) and (*s*) denote the liquid and solid phases, respectively, and *S* and *AS* represent the active sites capable of adsorbing adsorbate *A* and those sites occupied by *A* on the surface of the adsorbent, respectively.

Since the adsorption kinetics of Pb(II) ions on Cu-BTC MOFs agreed better with the pseudo second order model, one of the reasonable interpretations for such an observation is that the rate of adsorption in this case was being controlled by two limiting species: (a) the number of adsorbates near the adsorbent’s surface (not necessarily equal to the overall number of adsorbates in the bulk solution), and (b) the number of active adsorption sites on the adsorbent’s surface. The former was affected by the first adsorption step, namely, the transport of the adsorbates to the exterior of the adsorbents, while the latter was affected by the second adsorption step, namely, the binding of adsorbates to the adsorption sites. Therefore, both the transport and the binding steps were rate-limiting in the adsorption kinetics of Pb(II) ions on Cu-BTC MOFs. Since micropore surface areas in the Cu-BTC MOFs of this study accounted for the majority of the total surface areas, as shown in Table 2, pore diffusion rather than film diffusion is believed to have controlled the transport of Pb(II) ions in this case. Therefore, most transport must occur within the pores of Cu-BTC MOFs rather than their external surface. With regard to the fact that the number of active adsorption sites on the Cu-BTC’s surface also controls the rate of adsorption, it could be indicative of heterogeneous rather than homogenous adsorption occurring on the Cu-BTC MOFs. In other words, adsorption occurs only on the Cu-BTC’s surface with preferred sites and those sites become scarce when adsorption progresses. As a result, Pb(II) ions must travel further into the pores to reach those unoccupied preferred sites.

#### 2.2.2. Adsorption Equilibrium Study

From an equilibrium point of view, adsorption from a solution results in the continuous uptake of adsorbates until the concentration of adsorbates remaining in the solution is in equilibrium with that at the surface of adsorbents. In the literature, such an equilibrium is described in terms of an adsorption isotherm, in which the uptake of adsorbates per until weight of adsorbent, defined above as adsorption capacity (*q_e_*), is plotted against the concentration of adsorbates remaining in solution (*C_e_*) when equilibrium is reached. In the current study, measurements were made on a series of lead solutions with the initial concentration (*C*_0_) ranging from 10 mg/L to 1500 mg/L. After equilibrium was reached, *C_e_* was determined by AAS, from which *q_e_* was calculated, as described in Section 3.4. In many studies [11,12,14,19,21], however, most of the data collected for adsorption isotherms fell in the range of unchanged *q_e_*, which means that in those solution, adsorbents were saturated with adsorbates. This practice may cause some unintended bias toward the data points prior to saturation being reached if caution is not taken. The current study included the solutions in the range of 10–50 mg/L with an increment of 10 mg/L for adsorption measurements in addition to those with concentrations between 100 and 1500 mg/L. This assured that enough data in the low concentration range were included in the equilibrium study.

**Figure 6:**
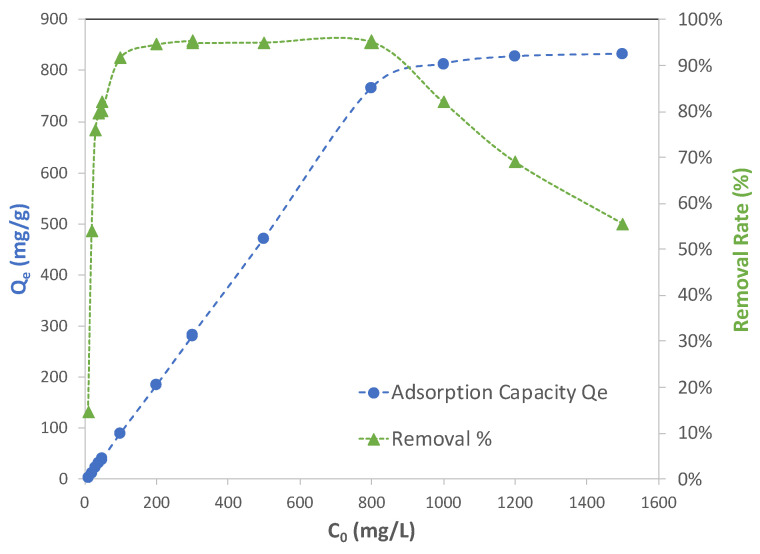
Adsorption capacity (blue dot) and removal rate (green triangle) of Pb(II) by Cu-BTC@STD from lead solution with various initial concentrations (*t* = 120 min).

Figure 6 illustrates how adsorption capacity (*q_e_*) and removal percent changed with the initial concentration of lead solution (*C*_0_). The blue dot curve in the figure shows that adsorption capacity started at a low value, meaning that Cu-BTC MOFs were not being fully utilized but increased until they reached a plateau after the initial concentration reached 800 mg/L. This indicates that at this concentration, Cu-BTC MOFs were close to maximizing their capacity of adsorbing Pb(II) ions. At concentrations higher than 800 mg/L, Cu-BTC MOFs left most of the excess Pb(II) ions unadsorbed. The adsorption performance of Cu-BTC MOFs could also be seen from the removal percent results, which are represented by the green triangle curve in Figure 6. The MOFs displayed the highest removal of near 95% for the solutions between 100 mg/L and 800 mg/L, and beyond 800 mg/L, the removal percent declined. Since Cu-BTC MOFs only reached their maximum capacity of adsorption at the concentration of 800 mg/L, this led to an expectation that the MOFs in the solutions with a lower concentration than 800 mg/L would carry a comparable level of capacity to that in the 800 mg/L solution. However, it is seen from Figure 6 that the removal percent started at only about 15% for the solutions of 10 mg/L and then increased until it starts to plateau at the concentration of 100 mg/L.

The equilibrium results shown in Figure 6 corroborated the interpretation made earlier from the kinetic data in terms of the two steps involved in adsorption, including the transport of adsorbates (mainly by pore diffusion) and the binding between adsorbate and adsorbent. The transport of adsorbates in solution is driven by a departure from an equilibrium condition, under which the concentration of adsorbates remaining in the bulk solution is equal to that at the surface of adsorbents. This is the “driving force” of the transport. On the other hand, when adsorbates travel in the fluid, passing through the pores inside the adsorbents until they find vacant adsorption sites, they also experience resistance. For given adsorbates, such a resistance force could be affected by the chemical and physical properties of adsorbents, such as the nature of their surface charges, their surface chemistry, as well as the geometry and the size of pores of the adsorbents. When adsorbents are porous, especially with micropores in the majority, pore diffusion rather than film diffusion is in control of the transport, as it was in the samples of the current study. In such cases, the resistance will become more prominent, and thus it must be taken into consideration when adsorption is being investigated. In simplifying analyses of such transport activity involving both driving and resistance forces, it is reasonably assumed that the driving force has a strong correlation with the concentration of adsorbates. Since the resistance is mainly affected by the property of adsorbents, it is justified to assume that it has negligible a correlation with the concentration of adsorbates.

**Figure 7:**
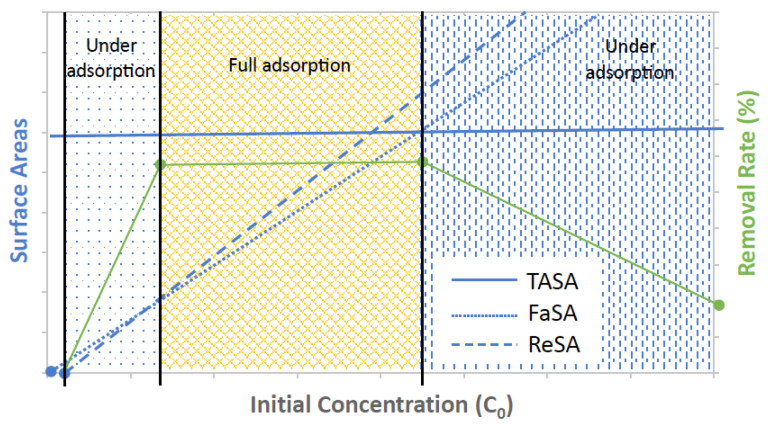
Schematic diagram of the change in reachable surface area (ReSA), full-adsorption surface area (FaSA), and total accessible surface area (TASA) with the initial concentration of adsorbates and illustration for how pore diffusion affects adsorbates being adsorbed on adsorbents.

Overall, the net effect between the driving force and the resistance force will result in adsorbates moving through the internal space of porous adsorbents and reaching the active sites for adsorption. To better illustrate how such a net effect on adsorbates determines adsorption, a schematic diagram is shown in Figure 7. The net effect on adsorbates is directly attributed to the amount of surface area (therefore the number of active adsorption sites) that adsorbates can reach, namely, reachable surface area (ReSA), as represented by a blue dashed line in the figure. As the initial concentration of adsorbates increases, so does the net effect. Therefore, the reachable surface area increases with concentration. At the low concentration range, however, the net effect is not strong enough to drive adsorbates to reach such an amount of surface areas to achieve full adsorption (as defined by the highest removal percent that the samples can achieve in this regard). The threshold for full adsorption is linked to the so-called full-adsorption surface area (FaSA), which is represented by a blue dotted line in Figure 7. The full-adsorption surface area is directly proportional to the initial concentration of adsorbates. Within the range of low concentrations, the reachable surface area (ReSA) is smaller than the full-adsorption surface area (FaSA), as illustrated in Figure 7. As a result, the removal percent within this range, as represented by the green line, is not able to reach its highest level. Such a range corresponded to 10–100 mg/L for Cu-BTC MOFs, as previously shown in Figure 6. For these solutions, pore diffusion clearly played a dominate role in the “under-adsorption” performance of the MOFs. After the concentration became high enough, which was 100 mg/L for Pb(II) adsorption on Cu-BTC, the net effect was then strong enough so that the reachable surface area surpassed the full-adsorption surface area. As expected, adsorption arrived at the highest rate. As the concentration continued to increase, adsorbates were capable of reaching more surface area in such a way as to maintain at the highest rate of adsorption. Therefore, “full adsorption” was used to characterize the performance within this range. For Pb(II) adsorption on Cu-BTC, this corresponded to the concentration range between 100 and 800 mg/L, as shown in Figure 6, where *q_e_* continued to rise, and the removal percent remained at the highest level. As the concentration moved even higher, however, adsorbates already used the total accessible surface area (TASA) existing in the adsorbents as represented by the blue solid line in Figure 7. Therefore, after that, the adsorption capacity plateaued, and the removal percent started to decline. This was observed for the adsorption of Pb(II) on Cu-BTC in the solution beyond 800 mg/L. Since the surface areas were directly related to the number of active adsorption sites, the “under-adsorption” performance within this concentration range was clearly due to the fact the availability of active sites limited adsorption in these solutions, which was different than the “under-adsorption” observed in the low concentration range, as mentioned earlier.

**Figure 8:**
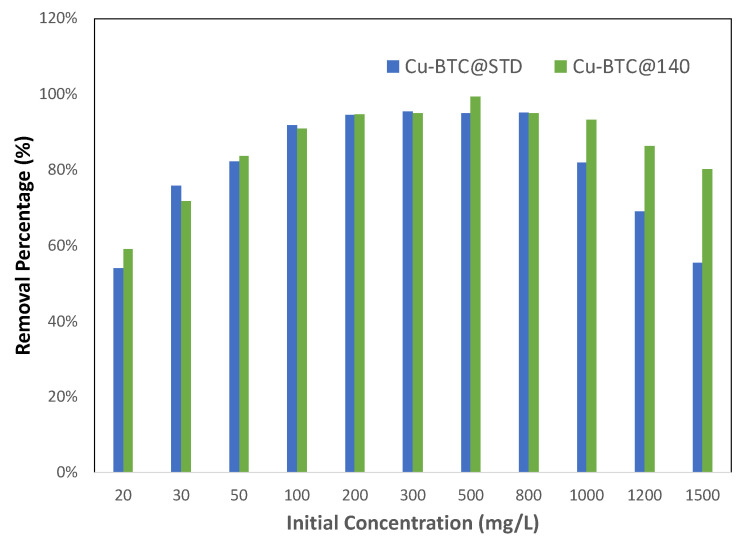
Effect of surface area on the removal % of Pb(II) by Cu-BTC MOFs from lead solutions with various initial concentrations (*t* = 120 min).

To further examine the role that the availability of active sites plays in adsorption, equilibrium measurements were also made to compare between two sets of Cu-BTC MOFs with different surface areas. The results are shown in Figure 8. As stated in Section 3, Cu-BTC@STD is a commercial product, while Cu-BTC@140 is our lab-made sample synthesized at 140 °C by using microwave method. As shown in Table 1, both samples contained a majority of micropores in their structure with similar pore sizes. However, their surface areas were quite different, with Cu-BTC@STD at 714 m^2^/g vs. Cu-BTC@140 at 1930 m^2^/g. With the Pb(II) concentration of 100 mg/L and below, as illustrated in Figure 8, Cu-BTC@140 showed a very similar adsorption performance to Cu-BTC@STD in terms of the removal percent, both of which climbed along with an increase in the concentration until it peaked at 100 mg/L. The reachable surface areas in both MOFs were comparable due to their similarity in pore dimensions, but they were lower than the full-adsorption surface areas for those concentrations. Such similar behavior between these two MOFs is consistent with the analysis made earlier, further indicative that the resistance experienced by adsorbates in the pore diffusion, rather than the number of active adsorption sites, played a determining role in the adsorption performance within this range of concentration. For the concentration between 100 and 800 mg/L, the reachable surface areas in both MOFs became larger than the full-adsorption surface areas, which means that adsorbates in both MOFs were able to reach enough surface area to be adsorbed so that their removal percent was maintained at a peak level. The difference in adsorption performance between these two MOFs appeared in the region beyond 800 mg/L. Cu-BTC@140 contained a larger total available surface area and thus more active adsorption sites than Cu-BTC@STD. As a result, the former was able to adsorb more Pb(II) ions, keeping its removal percent at the peak level until 1000 mg/L, while the latter already started to decline in its removal percent. After 1000 mg/L, a decrease in the removal percent in the Cu-BTC@140 sample also appeared, but such a decline occurred at a much slower pace than did the Cu-BTC@STD sample. Such an observation provided further corroborating evidence that the availability of active sites limits adsorption in the step where adsorbates attempt to bind to adsorbents.

**Figure 9:**
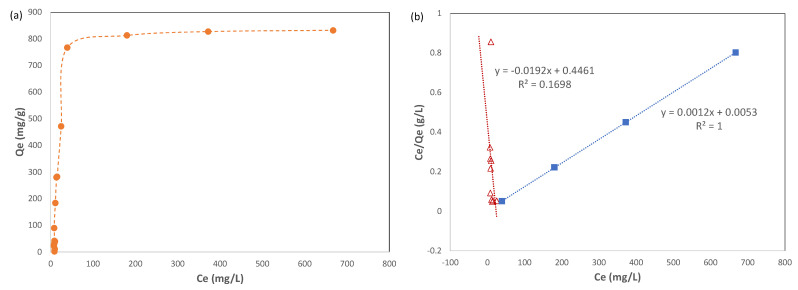

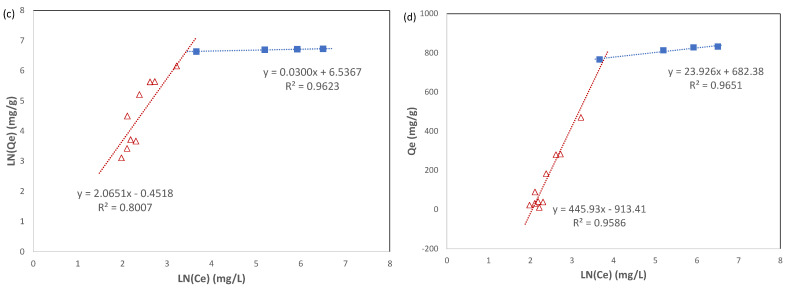
(**a**) Adsorption isotherm of Pb(II) on Cu-BTC@STD (*t* = 120 min) and its data fitting with the (**b**) Langmuir model, (**c**) Freundlich model, and (**d**) Temkin model. The red triangle curve represents the data from the acceleration stage of adsorption, and the blue square curve shows the slowdown stage of adsorption.

Equilibrium data for the adsorption of Pb(II) ions on Cu-BTC MOFs were also further studied by fitting them with numerical models. At the first, the adsorption isotherm of Pb(II) ions on Cu-BTC MOFs was plotted between the adsorption capacity (*q_e_*) against the equilibrium concentration of adsorbates (*C_e_*), and the results are shown in Figure 9a. As the equilibrium concentration increased, there was a sharp increase in the adsorption capacity at the initial stage (referred to as the “acceleration stage”), followed by a much slower second stage (“slowdown stage”). Two of the most-commonly used models for fitting isotherms are the Langmuir isotherm model [31] and the Freundlich isotherm model [32].

The Langmuir model assumes that the surface of adsorbents is homogeneous, that only a monolayer of adsorbates is formed on the surface of adsorbents, and that adsorbed molecules do not interact with one another. The linearized form of the Langmuir isotherm is given by Equation (4):

(4) $$\frac{C_{e}}{q_{e}}=\frac{1}{K_{L}q_{m}}+\frac{C_{e}}{q_{m}}$$

where *q_e_* and *C_e_* are the same quantities as defined previously, *K_L_* (L/mg) is the Langmuir constant that reflects the affinity of adsorbate and adsorbent, and *q_m_* (mg/g) is the maximum adsorption capacity.

Unlike the Langmuir model, the Freundlich model was established based on the assumption that multilayer adsorption occurs on a heterogeneous adsorbent surface. It also recognizes the interaction of adsorbed molecules. The linearized form of the Freundlich isotherm is given by Equation (5):

(5) $$\ln q_{e}=\ln K_{F}+\frac{1}{n}\ln C_{e}$$

where *K_F_* (unit depends on *n*) is the Freundlich constant, which is related to the adsorption capacity, and *n* is an empirical number related to the adsorption intensity.

A third numerical model, the Temkin model [33], was also used for the isotherm analysis in this study. Similar to the Freundlich model, this model recognizes multilayer adsorption and the interaction in the layer of adsorbed molecules. However, the Temkin model assumes that the intensity of such an interaction, expressed as heat of adsorption, decays linearly with increasing surface coverage instead of logarithmically, as implied in the Freundlich equation. The Temkin model is described by Equation (6):

(6) $$q_{e}=\frac{RT}{b_{T}}\ln A_{T}+\frac{RT}{b_{T}}\ln C_{e}$$

where *A_T_* (L/g) is the Temkin constant, related to the equilibrium binding constant corresponding to the maximum binding energy, and *b_T_* (J/mol) is the heat of adsorption.

The experimental adsorption isotherm data included in Figure 9a were fitted with the three numerical models, as described above, and the results are shown in Figure 9b–d. The data fitting was performed for each individual stage of the adsorption isotherm, the acceleration stage and the slowdown stage, instead of over the whole range. The calculated constants for each model and their coefficient of determination (R^2^) are summarized in Table 3.

In the acceleration stage, the coefficients of determination (R^2^) for the Freundlich model and the Temkin model were 0.8007 and 0.9586, respectively, which were superior to that for the Langmuir model, which was 0.1698. Therefore, the Freundlich model and the Temkin model were more satisfactory to describe this stage of adsorption. This supports the scenario that the uptake of Pb(II) ions on Cu-BTC MOFs is multilayer adsorption with preferential binding sites on the adsorbent surface. The fitting results also confirmed that Pb(II) ions in the adsorbed layer have interaction with one another as well as with Cu-BTC MOFs. Since the Temkin model was a better fit with the experiment data in the acceleration stage compared to the Freundlich model, it suggests to some degree that the heat of adsorption, indicative of the intensity of interaction among those adsorbed on Cu-BTC MOFs, decays linearly along with the increasing coverage of the adsorbents. As adsorbates are built up by layers, such an interaction continues to weaken until it does not result in a net gain between adsorbates being adsorbed and those leaving the adsorbents. The model-related constants are also included in Table 3. Because of the roughness of these models, however, caution has been taken for interpretating the physical meanings of these constants merely based on their magnitude, unless such claims could be verified by other methods.

In the slowdown stage of adsorption, the comparison among all three models showed that the Langmuir isotherm had the best fit to the experimental data, with the coefficient of determination (R^2^) at 1. This suggests that a monolayer of Pb(II) ions was formed during this stage of adsorption. However, it occurred to such a very limited extent that the adsorption capacity only increased lightly during this stage, as shown in Figure 9a. The maximum uptake capacity (*q_m_*) for Pb(II) ions derived from the Langmuir equation was 833.3 mg g^−1^, which was quite close to the experimental result as well as the one predicted from the kinetic model. In the literature [10,11,17,21], the maximum uptake capacity for the adsorption of Pb(II) ions on Cu-BTC MOFs ranges from 220 to 950 mg/g. The results in the current study showed that the Cu-BTC synthesized by the microwave method carried a high capacity for the adsorption of Pb(II) ions.

**Figure 10:**
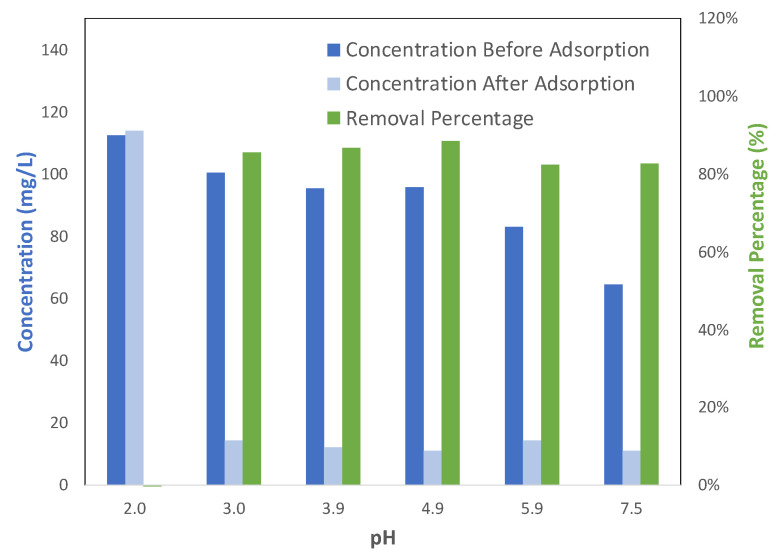
Removal rate of Pb(II) by Cu-BTC@STD from the solutions at different pH values (*C*_0_ = 100 mg/L, *t* = 120 min).

Most adsorption occurring in solution, especially with heavy metal ions being involved, is dependent on the pH of the solution, since pH not only affects the surface charge of adsorbents but also the form of heavy metal ions. Therefore, examining the effect of pH on adsorption could provide insight into the adsorption mechanism. Figure 10 shows how the adsorption of Pb(II) ions on Cu-BTC MOFs changed with various pH values of the solution. For pH < 3, there was little adsorption between Pb(II) ions and Cu-BTC MOFs. However, above this threshold value, the results showed that a significant amount of Pb(II) ions was adsorbed, and the removal % was comparable among all the solutions being studied. For transition divalent metals, such as Pb(II) in the current study, the most dominant species in their salt solutions are cations, such as Pb^2+^ and Pb(OH)^+^ [13,34], unless the solutions are adjusted to be basic. Positively charged hydronium ions (H_3_O^+^) also co-exist in the solution. Therefore, these metal cations may have to compete with hydronium ions for the preferential binding sites on the adsorbent. What is observed from Figure 10 validated such a scenario for the adsorption of Pb(II) ions on Cu-BTC MOFs. At the pH of 2, there was a large amount of hydronium ions existing in the solution. Hydronium ions have advantage over Pb(II) ions so that they occupy most of the active adsorption sites on Cu-BTC MOFs. When this happens, the surface charge of Cu-BTC might be also changed from being negative to positive. As a result, Pb(II) ions could be repulsed from, instead of being attracted to, the surface of the MOFs. That is why few of the Pb(II) ions were adsorbed at pH = 2. When the pH value was larger, however, the concentration of hydronium ions became lower. As a result, more of the preferential binding sites may have remained available for Pb(II) ions to be adsorbed to, and at the same time, the surface charge of Cu-BTC MOFs may also have remained negative, so that additional layers of Pb(II) ions could be adsorbed, even after hydronium ions were adsorbed. Therefore, the pH results further supported that there are preferential binding sites on the surface of Cu-TBC. As for the chemical nature of those preferential binding sites, it was reported in the literature [8,13] that they would be the negative regions at the carboxylic acid group on Cu-BTC MOFs, but this is beyond the scope of the current study.

## 3. Materials and Methods

### 3.1. Materials

Copper (II) nitrate trihydrate, Cu(NO_3_)_2_·3H_2_O (99–104%), and 1,3,5-benzenetricarboxylic acid or trimesic acid, H_3_BTC (>95%), were obtained from Sigma Aldrich (St. Louis, MO, USA). They were used as precursor materials to synthesize copper benzene-1,3,5-tricarboxylate MOFs without further purification. Lead (II) nitrate, Pb(NO_3_)_2_ (>99.0%), also purchased from Sigma Aldrich, was used to prepare lead stock solution.

### 3.2. Synthesis Procedures

In the current study, Cu-BTC MOFs were prepared via microwave synthesis using an Anton Paar Monowave 400 microwave reactor (Anton Paar USA Inc., Ashland, VA, USA). In a typical synthesis, 1.4496 g (6 mmol) of copper (II) nitrate trihydrate, 0.84056 g (4 mmol) of trimesic acid, and 15 mL of the 1:1 (*v*/*v*) ethanol and water mixture were added to a G30 vial with a magnetic stir bar and reacted for 10 min at temperatures ranging from 100 to 175 °C. The bright blue product was then vacuum filtered and washed three times with 10 mL of the 1:1 ethanol and water mixture. The washed product was dried overnight in an oven set to 150 °C. After completely drying, the samples were weighed and stored for further tests. The yields of Cu-BTC MOFs prepared at 140 and 160 °C were determined to be 32% and 86%, respectively. To simply the notation of Cu-BTC MOFs synthesized at a given temperature of X by the microwave method, they are denoted as Cu-BTC@X in this manuscript.

In addition, Basolite^®^ C 300, which is Cu-BTC MOFs produced by BASF, was acquired from Sigma Aldrich. In this manuscript, it is referred to as Cu-BTC@STD and was used for adsorption measurements without additional treatment.

### 3.3. Characterization Methods

All Cu-BTC samples were run for FTIR analysis with a Perkin-Elmer Spectrum Two (PerkinElmer Inc., Waltham, MA, USA). Powder X-ray diffraction analysis was conducted using a Panalytical X’Pert X-ray Diffractometer (Malvern Panalytical Ltd., Malvern, UK), with copper K_α_ radiation. A Micromeritics ASAP 2420 (Micromeritics Instrument Corp., Norcross, GA, USA) was employed to measure the nitrogen adsorption isotherms of the samples at −196 °C, which were used to determine the specific surface area and the pore size of the samples. Prior to measurement, samples were degassed under vacuum at 200 °C for 12 h. Multipoint BET calculations were performed at relative pressures (P/P0) in the range of 0.05–0.30.

### 3.4. Adsorption Measurements

The concentration of Pb(II) ions in the solution after adsorption experiments was measured by using a Shimadzu Atomic Absorption Spectrophotometer (AAS) AA 7000 (Shimadzu Corp., Kyoto, Japan). A lead stock solution of 2000 mg/L was first prepared by dissolving lead(II) nitrate in deionized water (DI). This stock solution was then used for serial dilutions for concentrations ranging from 1 mg L^−1^ to 1500 mg/L of lead. Then 10 mg of Cu-BTC MOFs was weighed accordingly before being placed in a clean 25 mL Erlenmeyer flask. In each trial, 10 mL of the appropriate lead solution was added to the sample flask. Trials at varying lead concentrations were put on a shaker for 2 h at room temperature and a rate of 300 rpm to ensure the MOFs were fully saturated. Trials were mixed at varying mix times ranging from 5 min to 120 min of mixing. After mixing, the MOFs were filtered out of the solution using a syringe filter. The lead solutions were then analyzed by AAS in triplicate using lead standards of 1, 5, 10, 15, and 20 mg/L to calibrate the instrument before every run. Additional trials were performed to analyze the pH effects on the adsorption performance of Cu-BTC MOFs. Then 0.1 M hydrochloric acid or 0.1 M sodium hydroxide was added dropwise to a 100 mg/L lead solution, and the pH of the solution was obtained between 2 and 7.5.

The adsorption capacity of Pb(II) ions at equilibrium (*q_e_*, mg/g) was calculated using the following equation:

(7) $$q_{e}=\frac{\left(C_{0}-C_{e}\right)V}{W}$$

where *C*_0_ (mg/L) is the initial concentration of the lead solution, *C*_e_ (mg/L) is the equilibrium concentration, *V* (L) is the volume of the solution, and *W* (g) is the mass of Cu-BTC MOFs. For the kinetic measurement, the capacity (*q_t_*, mg/g) at a given time *t* (mins) was calculated using a similar equation, where *C_t_*, the concentration at the time *t*, is in place for *C_e_*.

In addition, the removal efficiency was also calculated in terms of the percentage using the following equation:

(8) $$Removal\;\%=\frac{C_{0}-C_{e}}{C_{0}}\times 100\%\;\;\;\;\;\;\;\;\;\;\;\;\;\;\;\;\;\;\;\;\;\;\;\;\;\;\;\;\;\;\;\;\;$$

## 4. Conclusions

In the current study, effort was made to understand the mechanism for the adsorption of Pb(II) ions on Cu-BTC MOFs. This was achieved by examining their adsorption kinetic and equilibrium results. The kinetic data analysis lead to the findings that the limiting steps in the adsorption of Pb(II) ions on Cu-BTC are determined by (a) pore diffusion of Pb(II) ions and (b) the availability of the active sites on Cu-BTC MOFs. The equilibrium data analysis further confirmed the critical roles of these two steps in the adsorption of Pb(II) ions on Cu-BTC MOFs. The corresponding results revealed that micropores in the MOFs limit the diffusion of Pb(II) ions in the pores of Cu-BTC when the lead concentration is low. As a result, the removal percent of Pb(II) ions is not able to reach its highest level for the MOFs. When the concentration is high, however, the surface areas of the MOFs, which are proportional to the number of active adsorption sites on the MOFs, control their performance of adsorbing Pb(II) ions. These results could be used to improve adsorptive performance of Cu-BTC and other MOFs in wastewater treatment based on different conditions. For adsorption from the solutions with low concentrations of heavy metal ions, the focus should be place on choosing the proper pore size for the MOFs used for adsorption, while focus should be on the surface areas of the MOFs for those solutions with high concentrations.

## Figures and Tables

**Table 1:** Summary of textural properties of Cu-BTC samples.

Cu-BTC Sample	Specific Surface Area (m^2^/g)			Micropore Area Percentage (%)	Micropore Volume (cm^3^/g)	Pore Width (Å)
	BET Surface Area	Micropore Area	External Surface Area			
Cu-BTC@STD	714	633	81	88.7%	0.242	5.90 8.04
Cu-BTC@140	1930	1756	174	91.0%	0.673	8.58
Cu-BTC@160	1740	1586	154	91.1%	0.552	6.69 8.04

**Table 2:** Kinetic data fitting parameters of Pb(II) adsorption on Cu-BTC MOFs.

Initial Concentration (mg/L)	*q_e_* (mg/g) Experiment	Pseudo-First-Order Model			Pseudo-Second-Order Model		
		*q_e_* (mg/g)	*K*_1_ (min^−1^)	R^2^	*q_e_* (mg/g)	*K*_2_ (g/mg/min)	R^2^
100	91.98	78.58	0.2175	0.9902	93.46	0.01659	0.9952
1000	829.6	814.4	0.1071	0.9511	833.3	0.00048	0.9938

**Table 3:** Isotherm data fitting parameters of Pb(II) adsorption on Cu-BTC MOFs.

Adsorption Stage	Langmuir Model			Freundlich Model			Temkin Model		
	*q_m_* (mg/g)	*K_L_* (L/mg)	R^2^	*n*	*K_F_*	R^2^	*b_T_* (J/mol)	*A_T_* (L/g)	R^2^
Acceleration Stage	−52.08	−0.0430	0.1698	0.4842	0.6365	0.8007	5.649	0.1290	0.9586
Slowdown Stage	833.3	0.2264	1	33.33	690.0	0.9623	105.3	2.433 × 10^12^	0.9651

## Data Availability

Not applicable.

## Sample Availability

Samples of the compounds are available from the authors.
